# Motoric Cognitive Risk Syndrome and Traffic Incidents in Older Drivers in Japan

**DOI:** 10.1001/jamanetworkopen.2023.30475

**Published:** 2023-08-25

**Authors:** Satoshi Kurita, Takehiko Doi, Kenji Harada, Osamu Katayama, Masanori Morikawa, Chiharu Nishijima, Kazuya Fujii, Yuka Misu, Ryo Yamaguchi, Georg Von Fingerhut, Daisuke Kakita, Hiroyuki Shimada

**Affiliations:** 1 Department of Preventive Gerontology, Center for Gerontology and Social Science, Research Institute, National Center for Geriatrics and Gerontology, Obu, Japan; 2 Japan Society for the Promotion of Science, Tokyo, Japan; 3 Department of Medical Sciences, Medical Science Division, Graduate School of Medicine, Science and Technology, Shinshu University, Matsumoto, Japan

## Abstract

**Importance:**

To prevent motor vehicle collisions by older drivers, the increased risk of collisions should be considered early. Cognitive decline increases the risk of car collisions. Motoric cognitive risk syndrome (MCR), characterized by the presence of cognitive concerns and slow gait, can be assessed conveniently and is useful to assess the risk of dementia.

**Objective:**

To examine the association between MCR assessment findings and car collisions among older drivers in Japan.

**Design, Setting, and Participants:**

This cross-sectional study used data from a community-based cohort study, the National Center for Geriatrics and Gerontology-Study of Geriatric Syndromes, conducted in Japan from 2015 to 2018. Participants were community-dwelling older adults aged at least 65 years. Data were analyzed from February to March 2023.

**Exposure:**

MCR was defined as having subjective memory concerns (SMC) and slow gait. Participants were classified into 4 groups: no SMC or slow gait, only SMC, only slow gait, and MCR.

**Main Outcomes and Measures:**

Participants were asked about the experience of car collisions during the last 2 years and near-miss traffic incidents during the previous year through face-to-face interviews. Odds of experiencing a collision or near-miss traffic incident were assessed using logistic regression.

**Results:**

Among a total of 12 475 participants, the mean (SD) age was 72.6 (5.2) years, and 7093 (56.9%) were male. The group with only SMC and the group with MCR showed a higher proportion of both car collisions and near-miss traffic incidents than the other groups (adjusted standardized residuals > 1.96; *P* < .001). Logistic regression analysis showed the only SMC and MCR groups had increased odds of car collisions (only SMC group: odds ratio [OR], 1.48; 95% CI, 1.27-1.72; MCR group: OR, 1.73; 95% CI, 1.39-2.16) and near-miss traffic incidents (only SMC group: OR, 2.07; 95% CI, 1.91-2.25; MCR group: OR, 2.13; 95% CI, 1.85-2.45) after adjusting for confounding factors. After stratifying MCR assessments by objective cognitive impairment, significant associations were still observed. In the only slow gait group, objective cognitive impairment was associated with increased odds of car collisions (OR, 1.96; 95% CI, 1.17-3.28).

**Conclusions and Relevance:**

In this cross-sectional study of community-dwelling older drivers in Japan, SMC and MCR were associated with car collisions and near-miss traffic incidents independent from objective cognitive impairment. Future studies should examine the mechanism of these associations in more detail.

## Key Points

**Question:**

What is the association of motoric cognitive risk syndrome (MCR) with traffic incidents among older drivers?

**Findings:**

This cross-sectional study included 12 475 drivers in Japan aged at least 65 years. Drivers with subjective memory concerns (SMC) or MCR had significantly increased odds of experiencing of car collisions compared with drivers with no SMC or MCR, regardless of objective cognitive impairment.

**Meaning:**

This cross-sectional study found that older drivers with SMC or MCR had increased odds of experiencing car collisions.

## Teaser

This cross-sectional study examines the associations of motoric cognitive risk syndrome with odds of motor vehicle collisions and near-miss traffic incidents among older drivers in Japan.

## Introduction

Driving a car significantly correlates with life-space mobility in older adults.^1,2^ It is essential for older adults to maintain their independence, including meeting friends or family, traveling, enjoying entertainment, or shopping.^3,4^ However, aging causes perceptual and cognitive declines related to vision, hearing and vibration detection, attention, and speed of processing and responding, which are relevant to driving safely.^5^ Recent studies have reported that the severity of car collisions increases with age.^6,7^ To prevent car collisions in older adults, the increased risk of collisions associated with age should be evaluated early.

Driving performance is affected by cognitive function,^5,8^ especially executive function, attention, and processing speed.^9,10,11,12^ In some countries, including Japan, neuropsychological tests to assess cognitive impairment are required for older drivers when renewing their driver’s license. Motoric cognitive risk syndrome (MCR), identified by the presence of subjective memory concerns (SMC) and slow gait,^13^ has been associated with decreased processing speed and executive function^14^ and an increased risk of developing dementia or disability.^13,15^ Although assessing MCR is convenient and can be performed without the need for expert staff, the association between MCR and car collisions has not been examined. If there is an association between MCR and the risk of car collisions, MCR assessment may lead to an awareness of the increased risk at an early stage. Therefore, the aim of this study is to examine the association between MCR assessment and car collisions.

## Methods

This cross-sectional study was approved by the National Center for Geriatrics and Gerontology research ethics committee. This study was conducted according to the Declaration of Helsinki guidelines. All participants provided written informed consent. This study followed the Strengthening the Reporting of Observational Studies in Epidemiology (STROBE) reporting guideline.

### Participants

This study used a data set from the National Center for Geriatrics and Gerontology—Study of Geriatric Syndromes (NCGG-SGS). The NCGG-SGS is a community-based cohort study to establish a screening system for geriatric syndromes and validate evidence-based interventions for their prevention, which have been described elsewhere.^16^ The NCGG-SGS data set in this study contained the data of 19 598 community-dwelling older adults aged at least 65 years recruited from Obu, Takahama, Tokai, and Toyoake in Aichi Prefecture, Japan. The surveys were conducted from 2015 to 2017 (in Obu and Takahama) and 2017 to 2018 (in Toyoake and Tokai). For this study, participants were excluded if they did not drive (5826 participants), had a self-reported basic activity of daily living disability (10 participants), had a medical history that included stroke and dementia (772 participants), had a general cognitive impairment (Mini-Mental State Examination score <21; 130 participants),^17^ or had missing data for any of the variables used in our study (385 participants). A total of 12 475 participants were included in the analysis.

### Measurement

#### Motoric Cognitive Risk Syndrome

MCR was identified by having subjective memory concerns (SMC) and slow gait. In the assessment of SMC, we used several questions (in Japanese). One question uses a standardized memory loss question from the Geriatric Depression Scale: *do you feel you have more problems with memory than most?*^18^ A positive response (yes) on this question was identified as SMC. In addition, we also assessed SMC using the Cambridge Mental Disorders of the Elderly Examination questionnaire^19^ and the Subjective Memory Complaints scale, as used in previous studies^20,21^: *do you have any difficulty with your memory*,* do you forget where you have left things more than you used to*,* do you forget the names of close friends or relatives*, and *do other people find you forgetful?* At least 1 positive response to any of these 5 questions was identified as SMC.

Gait speed was assessed by walking on a 6.4-m flat, straight surface at a comfortable pace. Two markers were set in the middle to indicate a 2.4-m walking path to measure gait speed, and 2-m sections were set before and after passing the 2.4-m path to ensure a comfortable pace by the time they finished. Walking time was measured in seconds using a sensor, and the participants’ walking speed was calculated as meters per second. Slow gait was defined as −1.0 SD or lower below age- and sex- appropriate mean values established in the NCGG-SGS database. Using the assessment of SMC and slow gait, the participants were categorized into no SMC or slow gait (hereafter, *robust*), only SMC, only slow gait, or MCR groups.

#### Car Collisions and Near-Miss Traffic Incidents

As the primary outcome, the experience of car collisions was assessed using the following interview question: *do you have a history of any car collision during the last 2 years?* Participants who had a history of any car collisions were asked details about the incidents. Multiple answers could be selected from the following: (1) collision occurred when you were driving and involved persons on the road; (2) collision occurred when you were driving, and there was damage, such as property damage; (3) collision involved another car, and you were culpable for less than half of the fault; (4) collision involved another car, and you were culpable for more than half of the fault; and (5) others. We assessed the experience of car collisions overall and of car collisions excluding those that were less than half of driver’s fault.

As the secondary outcome, experiences of near-miss traffic incidents were assessed using questions about 12 situations while driving in the previous year. The participants were asked to answer yes or no about the following 12 experiences: (1) when going from a stop line, I almost hit someone coming from a different direction; (2) when attempting to turn right, I almost hit a car coming straight on; (3) when attempting to turn right, I almost hit a pedestrian or bicycle; (4) when attempting to turn left, I almost hit a pedestrian or bicycle; (5) I drifted greatly into the oncoming lane and almost collided head-on with another vehicle; (6) when changing lanes, I almost collided with another vehicle; (7) I almost collided with a vehicle in front of me; (8) I almost struck a bicycle, motorcycle, other vehicle I was overtaking or passing; (9) I made a mistake while stepping on the accelerator or brake; (10) when starting on a hill, I almost hit another vehicle or obstacle (including living things); (11) when backing up to park, I almost hit another vehicle or obstacle (including living things); and (12) when I entered a store parking lot from the road, I almost ran up on the curb of the sidewalk. We identified participants who had 1 or more experiences of near-miss incidents while driving.

#### Confounding Factors

Confounding factors were selected, referring to the factors of car collisions and dementia reported by previous reviews.^5,22^ In addition to the sociodemographic data (age, sex, and education years), medical (eye disease, hearing difficulty, and the number of medications used) and lifestyle (sleep duration, excessive daytime sleepiness, daily mean driving time) information were collected through a face-to-face interview. Eye diseases were assessed by the presence of glaucoma, cataract, or other diseases. Hearing difficulty was assessed by a question from the Hearing Handicap Inventory for the Elderly Screening Version (HHIE-S): do you have difficulty hearing when someone speaks in a whisper? (possible answers include yes, sometimes, or no).^23^ Participants who used 5 or more medications were identified as having polypharmacy.^24^ Sleep duration was categorized into 3 levels (≥7 hours, 6.0-6.9 hours, or <6 hours per night), referring to the literature on acute sleep deprivation and culpable motor vehicle collision involvement.^25^ Excessive daytime sleepiness was assessed using the question *how often do you experience daytime sleepiness that causes difficulty staying awake?*, with the following options in response: 3 or more days a week, 1 or 2 days a week, less than 1 day a week, and never. These responses were divided into 1 or more days in a week and less than a day a week. The mean daily driving time for a week was calculated as self-reported driving days in a week times driving time in a day, divided by 7. Sleep duration was calculated by the difference between self-reported usual sleep and wake times.

Objective cognitive impairment (OCI) was assessed by neuropsychological tests using the National Center for Geriatrics and Gerontology-Functional Assessment Tool, which has been described elsewhere.^26^ Four cognitive domain tests were conducted: memory (word list memory–I [immediate recognition] and word list memory–II [delayed recall]), attention (an electronic tablet version of the Trail Making Test [TMT] part A), executive function (an electronic tablet version of the TMT part B), and processing speed (an electronic tablet version of the Symbol Digit Substitution Test). Participants who returned results lower than the standardized thresholds for 1 or more tests in the Functional Assessment Tool were defined as having OCI.

### Statistical Analysis

The differences in participants’ characteristics among the robust, only SMC, only slow gait, and MCR groups were examined using 1-way analysis of variance and Pearson χ^2^ test for discrete variables. The differences in the outcomes among the 4 groups were also examined using Pearson χ^2^ test. In the χ^2^ test, adjusted standardized residuals (ASRs) were confirmed as either greater than 1.96 or less than −1.96 to identify specific cells. The associations between the outcomes and MCR assessment were examined using binomial logistic regression models in a crude model and a fully adjusted model for all covariates. Odds ratios (ORs) and 95% CIs referring to the robust group were calculated. In addition, to examine the interactions with OCI in the associations between MCR assessment and the outcomes, we categorized the participants into 8 groups by MCR assessment times the presence of OCI. The associations among the 8 groups and outcomes were similarly examined using binomial logistic regression models (with to the robust group without OCI as the reference group) in the fully adjusted model. All analyses were conducted using SPSS software version 25 (IBM). The level of statistical significance was set at *P* < .05 for all analyses, and tests were 2-sided. Data were analyzed from February to March 2023.

## Results

A total of 12 475 older drivers (mean [SD] age, 72.6 [5.2] years; 7093 [56.9%] male) were included in the study. Participants had a mean (SD) of 12.0 (2.4) years of education. Participant characteristics are summarized in Table 1. Stratified by cognitive scores, there were 3856 participants (30.9%) in the robust group, 6889 participants (55.2%) in the only SMC group, 557 participants (4.5%) in the only slow gait group, and 1173 participants (9.4%) in the MCR group. While the only slow gait and MCR groups showed a higher proportion of OCI than the other groups (ASR > 1.96; *P* < .001), the only SMC and MCR groups showed a higher proportion of eye diseases and hearing difficulty than the other groups (ASR > 1.96; *P* < .001). The only SMC group and MCR group also showed a higher proportion of excessive daytime sleepiness than the other groups (ASR > 1.96; *P* < .001).

**Table 1.** Participant Characteristics

	Participants, No. (%)					*P* value ^a^
Characteristic	Total (N = 12 475)	Robust (n = 3856)	Only SMC (n = 6889)	Only slow gait (n = 557)	MCR (n = 1173)	
Age, mean (SD), y	72.6 (5.2)	72.3 (5.0)	72.8 (5.3)	71.5 (5.0)	72.4 (5.1)	<.001^b^
Sex						
Male	7093 (56.9)	2201 (57.1)	3916 (56.8)	290 (52.1)	686 (58.5)	.09
Female	5382 (43.1)	1655 (42.9)	2973 (43.2)^c^	267 (47.9)	487 (41.5)	
Years of education, mean (SD), No.	12.0 (2.4)	12.1 (2.4)	12.0 (2.4)	11.6 (2.2)	11.7 (2.5)	<.001
Normal gait speed, mean (SD), m/s	1.17 (0.22)	1.24 (0.18)	1.22 (0.18)	0.86 (0.12)	0.85 (0.12)	<.001
Eye diseases	4134 (33.1)	1164 (30.2)^d^	2388 (34.7)^c^	172 (30.9)	410 (35.0)	<.001
Hearing difficulty						
No	6116 (49.0)	2297 (59.6)^c^	2935 (42.6)^d^	340 (61.0)^c^	544 (46.4)	<.001
Sometimes	4176 (33.5)	1145 (29.7)^d^	2502 (36.3)^c^	160 (28.7)^d^	369 (31.5)	
Yes	2183 (17.5)	414 (10.7)^d^	1452 (21.1)^c^	57 (10.2)^d^	260 (22.2)^c^	
Receiving ≥5 medications	2684 (21.5)	715 (18.5)^d^	1469 (21.3)	153 (27.5)^c^	347 (29.6)^c^	<.001
Sleep duration, h/night						
≥7.0	5998 (48.1)	1895 (49.1)	3279 (47.6)	260 (46.7)	564 (48.1)	<.001
6.0-6.9	4036 (32.4)	1300 (33.7)^c^	2225 (32.3)	185 (33.2)	326 (27.8)^d^	
<6.0	2441 (19.6)	661 (17.1)^d^	1385 (20.1)	112 (20.1)	283 (24.1)^c^	
Excessive daytime sleepiness	1807 (14.5)	349 (9.1)^d^	1159 (16.8)^c^	64 (11.5)^d^	235 (20.0)^c^	<.001
Driving time, mean (SD), min/d	50.5 (54.0)	52.5 (56.7)	48.5 (47.4)	56.1 (80.5)	53.2 (64.2)	<.001
Objective cognitive impairment						
Any	2756 (22.1)	734 (19.0)^d^	1456 (21.1)^d^	154 (27.6)^c^	412 (35.1)^c^	<.001
Word memory^b^	1281 (10.3)	335 (8.7)^d^	662 (9.6)^d^	69 (12.4)	215 (18.3)^c^	<.001
Trail Making Test–part A^b^	974 (7.8)	245 (6.4)^d^	502 (7.3)^d^	73 (13.1)^c^	154 (13.1)^c^	<.001
Trail Making Test–part B^b^	887 (7.1)	222 (5.8)^d^	461 (6.7)^d^	53 (9.5)^c^	151 (12.9)^c^	<.001
Symbol Digit Substitution test^b^	648 (5.2)	141 (3.7)^d^	306 (4.4)^d^	50 (9.0)^c^	151 (12.9)^c^	<.001

Abbreviations: MCR, motoric cognitive risk syndrome; SMC, subjective memory concerns.

^a^ Continuous variables between groups were assessed using 1-way analysis of variance. Category variables between groups were compared using Pearson χ^2^ test.

^b^ Percentage of those who returned results lower than the standardized thresholds in each test.

^c^ Adjusted standardized residual was greater than 1.96.

^d^ Adjusted standardized residual was less than −1.96.

**Figure 1.**
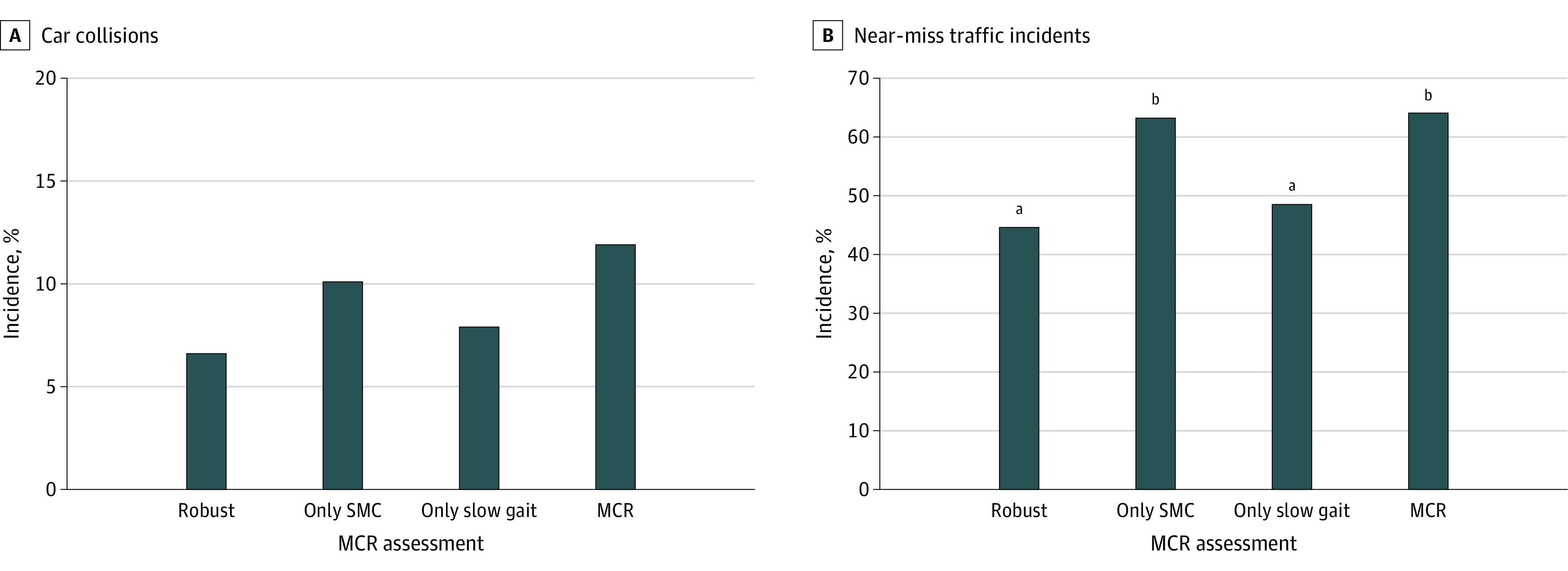
Comparison of Car Collisions and Near-Miss Traffic Incidents Among Older Drivers in Japan by Motoric Cognitive Risk Syndrome (MCR) Assessment Comparisons were assessed using χ^2^ test. MCR indicates motoric cognitive risk syndrome; SMC, subjective memory complaints. ^a^Adjusted standardized residual was greater than 1.96 (*P* < .05). ^b^Adjusted standardized residual was less than −1.96 (*P* < .05).

A comparison of car collisions and near-miss traffic incidents by MCR assessment is presented in Figure 1. In both car collisions and near-miss traffic incidents, the only SMC and MCR groups showed higher incidence rates than the other groups (ASR > 1.96; *P* < .001). These tendencies were consistent after excluding collisions that were less than half the older driver’s fault and each item of near-miss traffic incidents (eFigure 1 and eFigure 2 in Supplement 1).

The associations of MCR assessment with car collisions and near-miss traffic incidents from the logistic regression models are summarized in Table 2. The only SMC and MCR groups had increased odds of car collisions (only SMC group: OR, 1.48; 95% CI, 1.27-1.72; MCR group: OR, 1.73; 95% CI, 1.39-2.16) and near-miss traffic incidents (only SMC group: OR, 2.07; 95% CI, 1.91-2.25; MCR group: OR, 2.13; 95% CI, 1.85-2.45) after adjusting for confounding factors. Similar results were observed after excluding collisions that were less than half the older driver’s fault (only SMC group: OR, 1.71; 95% CI, 1.43-2.04; MCR group: OR, 2.06; 95% CI, 1.60-2.65) (eTable in Supplement 1). The slow gait group did not have significant associations with these outcomes.

**Table 2.** Logistic Regression Model for the Associations of MCR With Car Collisions and Near-Miss Traffic Incidents

	Car collisions				Near-miss traffic incidents				
	Crude		Adjusted		Crude		Adjusted		
	OR (95% CI)	*P* value	OR (95% CI)	*P* value	OR (95% CI)	*P* value	OR (95% CI)	*P* value
MCR assessment								
Robust	1 [Reference]	NA	1 [Reference]	NA	1 [Reference]	NA	1 [Reference]	NA
Only SMC	1.58 (1.36-1.84)	<.001	1.48 (1.27-1.72)	<.001	2.14 (1.98-2.32)	<.001	2.07 (1.91-2.25)	<.001
Only slow gait	1.21 (0.86-1.68)	.27	1.17 (0.83-1.63)	.368	1.17 (0.98-1.40)	.082	1.19 (0.99-1.43)	.06
MCR	1.91 (1.53-2.37)	<.001	1.73 (1.39-2.16)	<.001	2.21 (1.93-2.54)	<.001	2.13 (1.85-2.45)	<.001
Age, per 1-y increase	NA	NA	0.99 (0.98-1.01)	.243	NA	NA	0.98 (0.98-0.99)	<.001
Male sex	NA	NA	0.91 (0.80-1.04)	.17	NA	NA	1.52 (1.40-1.64)	<.001
Educational year	NA	NA	1.04 (1.01-1.07)	.003	NA	NA	1.03 (1.01-1.04)	.002
Eye disease	NA	NA	1.16 (1.01-1.32)	.04	NA	NA	1.07 (0.98-1.16)	.13
Hearing difficulty								
No	NA	NA	1 [Reference]	NA	1 [Reference]	NA	1 [Reference]	NA
Sometimes	NA	NA	1.18 (1.03-1.36)	.020	NA	NA	1.25 (1.15-1.36)	<.001
Yes	NA	NA	1.35 (1.14-1.6)	<.001	NA	NA	1.44 (1.30-1.61)	<.001
Medication use (ref: <5)	NA	NA	1.07 (0.92-1.25)	.36	NA	NA	1.05 (0.96-1.16)	.29
Sleep duration, h/night								
≥7	NA	NA	1 [Reference]	NA	1 [Reference]	NA	1 [Reference]	NA
6.1-6.9	NA	NA	1.18 (1.03-1.37)	.021	NA	NA	1.14 (1.04-1.24)	.003
≤6	NA	NA	1.32 (1.12-1.55)	.001	NA	NA	1.07 (0.97-1.19)	.16
Excessive daytime sleepiness^a^	NA	NA	1.32 (1.12-1.54)	.001	NA	NA	1.34 (1.21-1.50)	<.001
Driving time, per 1-min/d increase	NA	NA	1.00 (1.00-1.00)	<.001	NA	NA	1.00 (1.00-1.00)	<.001
Cognitive impairment	NA	NA	1.11 (0.96-1.29)	.15	NA	NA	0.91 (0.83-0.99)	.04

Abbreviations: MCR, motoric cognitive risk syndrome; NA, not applicable; OR, odds ratio; SMC, subjective memory concerns.

^a^ The reference group was participants who experienced daytime sleepiness less than 1 day per week.

**Figure 2.**
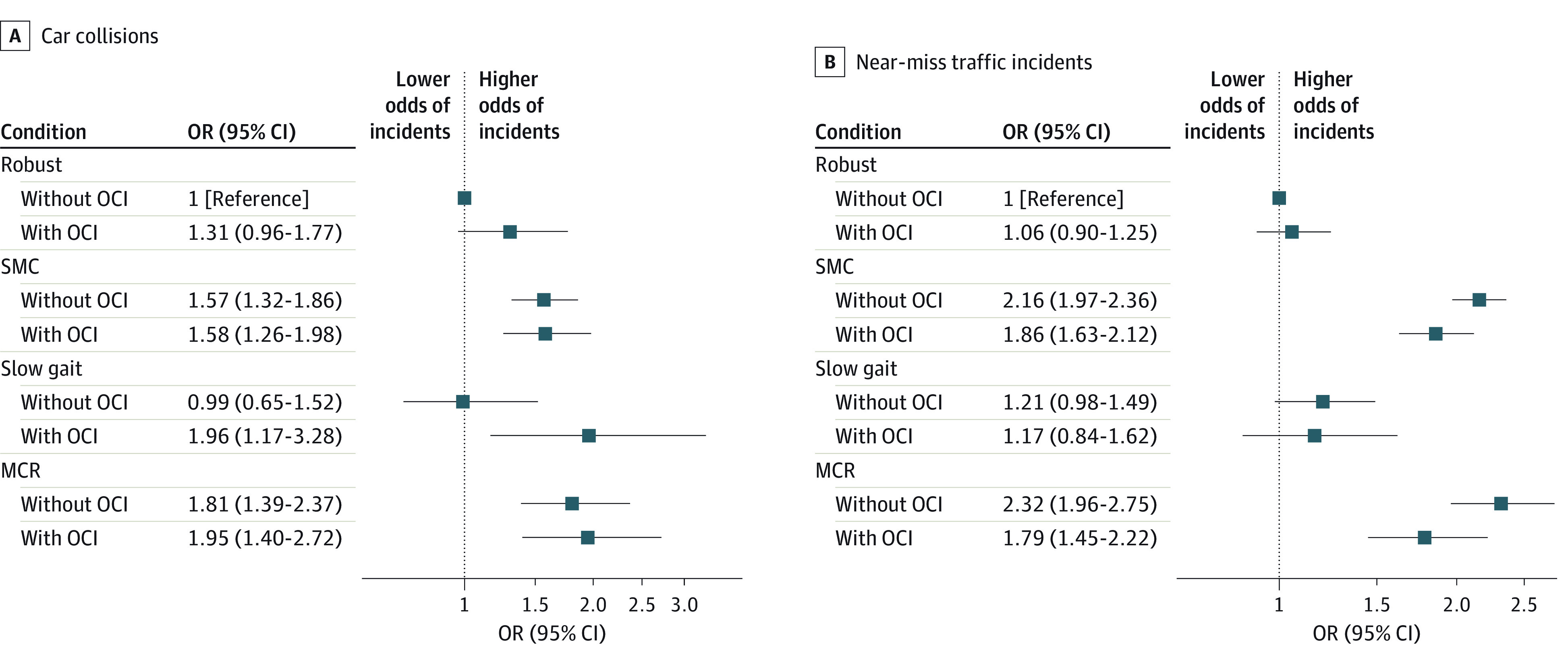
Interactions Between Motoric Cognitive Risk Syndrome (MCR) Assessment and Objective Cognitive Impairment (OCI) on the Associations With Car Collisions and Near-Miss Traffic Incidents Odds ratios (ORs) were adjusted for age, sex, educational year, eye diseases, hearing difficulty, medication use, sleep duration, excessive daytime sleepiness, and driving time. MCR indicates motoric cognitive risk syndrome; OCI, objective cognitive impairment; SMC, subjective memory concerns.

After stratifying MCR assessment by OCI, the only SMC and MCR groups had increased odds of car collisions and near-miss traffic incidents regardless of OCI (Figure 2A and B). In the only slow gait group, participants with OCI had increased odds of car collisions (OR, 1.96; 95% CI, 1.17-3.28) (Figure 2A). These tendencies were consistent after excluding car collisions that were less than half the older driver’s fault (eFigure 3 in Supplement 1).

## Discussion

This cross-sectional found that older drivers in Japan with SMC or MCR had higher odds of car collisions and near-miss traffic incidents. Significant associations with car collisions were also observed when excluding collisions that were less than half the driver’s fault. Except for the only slow gait group, these findings were consistent even after stratification by the presence of OCI.

Older drivers with SMC or MCR were inclined to experience car collisions or near-miss traffic incidents regardless of OCI. OCI, especially in executive function, attention, and processing speed, has been reported to be associated with car collisions.^9,10,11,12^ Our findings suggest that not only MCR but also SMC were factors associated with car collisions. In a large cohort study, the risk of cognitive impairment and dementia by MCR assessment was the highest in the MCR group compared with the only slow gait group and only SMC group.^27^ At the same time, MCR has been associated with several other diseases^28^ and an increased risk of old age–related poor outcomes, including frailty, falls, and disability.^29^ Because MCR is an unhealthy assessment in various aspects, the mechanism of the association between MCR and car collisions could be explained by factors other than cognitive function.

Participant characteristics in this study may partly explain the associations of SMC and MCR with car collisions. There were no obvious differences in self-reported sleep duration among participants with MCR, but the only SMC and MCR groups had higher proportions of excessive daytime sleepiness than the other groups. This result was in line with the association between sleep concerns and SMC reported by other cross-sectional studies,^30,31,32^ and sleepiness was reported as an independent risk factor associated with car collisions by a systematic review and meta-analysis.^25,33^ Although the causal relationship between sleep concerns and SMC has not been clarified, sleep concerns may increase the risk of not only car collisions but also SMC. In addition, the only SMC and MCR groups had more eye diseases and hearing difficulties than the other groups. In particular, hearing difficulty was significantly different among the MCR assessments. Previous studies have reported that hearing impairment was associated with self-reported car collisions in the past year^34^ and worse driving performance.^35^ Hearing impairment may be further problematic when accompanied by vision impairment.^36^ Although there is a lack of evidence concerning the associations of SMC and MCR with sensory disorders, sensory disorders may also be risk factors of SMC, MCR, or a preclinical stage of dementia,^13,22,37^ because hearing and visual impairment are risk factors associated with dementia.^22^ Future research should examine the influence of hearing and visual impairment on SMC and MCR in older adults.

The only slow gait group showed a relatively higher percentage of OCI than the other groups but did not have significantly increased odds of car collisions and near-miss traffic incidents. Compared with the robust group without OCI, the slow gait with OCI group had increased odds of car collisions. These findings suggested slow gait speed may not be a risk factor associated with car collisions in older drivers as long as there is no OCI. Because physical frailty is associated with car collisions,^38^ a comprehensive evaluation of frailty or MCR may be more suitable to estimate the risk of car collisions rather than measuring only gait speed.

### Strengths and Limitations

A strength of this study is that it is the first study to examine the association between MCR assessment and car collisions, to our knowledge. We were able to examine the association by targeting large numbers of older drivers using extensive cohort data.

This study has some limitations. First, the outcomes data were collected by a self-report method, and there may be recall bias. However, the credibility of our findings was compensated by multiple results, since the experiences of car collisions and near-miss traffic incidents among the 4 groups showed similar distribution: the MCR group was followed by the only SMC group, the only slow gait group, and the robust group. The proportion of 12 items of near-miss traffic incidents among the 4 groups also fell within expected bounds. Second, this study has a cross-sectional study design, and the causal relationships of MCR assessment with car collisions are unknown. This study design also could not assess whether the participant had MCR at the time of the car collisions and near-miss traffic incidents. Third, the excluded missing data or residual confounders after grouping by MCR assessment combined with the presence of OCI may have influenced the results.

## Conclusions

In this cross-sectional study of community-dwelling older drivers in Japan, SMC and MCR were associated with car collisions and near-miss traffic incidents independent from OCI. To increase the generalizability and examine the external validity of these findings, further studies in different settings are needed. The mechanism of these associations is also an issue for future studies.

## Notes

Supplement 1. **eTable.** Logistic Regression Model for the Associations of MCR Assessment Findings With Car Collisions Except Collisions That Were Less Than Half of Driver’s Fault **eFigure 1.** Comparison of Car Collisions Except Collisions That Were Less Than Half of Driver’s Fault Among MCR Assessment Using χ^2^ Test **eFigure 2.** Comparison of Each Item of Near-Miss Traffic Incidents Among MCR Assessment Using χ^2^ Test **eFigure 3.** Odds Ratios of Interactions Between MCR Assessment Findings and OCI on the Associations With Car Collisions Except Those That Were Less Than Half of Driver’s Fault

Supplement 2.
Data Sharing Statement
