# Supplementary material for: Motoric Cognitive Risk Syndrome and Traffic Incidents in Older Drivers in Japan
Source: JAMA Netw Open. 2023 Aug 25;6(8):e2330475. doi: 10.1001/jamanetworkopen.2023.30475 (PMC10457720; doi:10.1001/jamanetworkopen.2023.30475)
Supplement: Supplement 1. — eTable. Logistic Regression Model for the Associations of MCR Assessment Findings With Car Collisions Except Collisions That Were Less Than Half of Driver’s Fault eFigure 1. Comparison of Car Collisions Except Collisions That Were Less Than Half of Driver’s Fault Among MCR Assessment Using χ2 Test eFigure 2. Comparison of Each Item of Near-Miss Traffic Incidents Among MCR Assessment Using χ2 Test eFigure 3. Odds Ratios of Interactions Between MCR Assessment Findings and OCI on the Associations With Car Collisions Except Those That Were Less Than Half of Driver’s Fault [file jamanetwopen-e2330475-s001.pdf]

Supplemental Online Content

Kurita S, Doi T, Harada K, et al. Motoric cognitive risk syndrome and traffic incidents in older drivers in Japan. *JAMA Netw Open*. 2023;6(8):e2330475. doi:10.1001/jamanetworkopen.2023.30475

eTable. Logistic Regression Model for the Associations of MCR Assessment Findings With Car Collisions Except Collisions That Were Less Than Half of Driver's Fault
eFigure 1. Comparison of Car Collisions Except Collisions That Were Less Than Half of Driver's Fault Among MCR Assessment Using χ^2 Test
eFigure 2. Comparison of Each Item of Near-Miss Traffic Incidents Among MCR Assessment Using χ^2 Test
eFigure 3. Odds Ratios of Interactions Between MCR Assessment Findings and OCI on the Associations With Car Collisions Except Those That Were Less Than Half of Driver's Fault

This supplemental material has been provided by the authors to give readers additional information about their work.

© 2023 Kurita S et al. *JAMA Network Open.*

eTable. Logistic Regression Model for the Associations of MCR Assessment Findings With Car Collisions Except Collisions That Were Less Than Half of Driver's Fault

	Traffic violent			
	Crude		Adjusted	
	OR (95% CI)	p	OR (95% CI)	p
MCR assessment (ref: robust)				
Only SMC	**1.84 (1.54–2.19)**	**<0.001**	**1.71 (1.43–2.04)**	**<0.001**
Only slow gait	1.33 (0.91–1.96)	0.142	1.27 (0.87–1.88)	0.218
MCR	**2.30 (1.80–2.94)**	**<0.001**	**2.06 (1.60–2.65)**	**<0.001**
Age			0.99 (0.98–1.01)	0.491
Sex (ref: Female)			0.91 (0.78–1.05)	0.202
Educational year			1.04 (1.01–1.07)	0.015
Eye diseases (ref: No)			1.12 (0.96–1.30)	0.142
Hearing difficulty (ref: No)				
Sometimes			1.22 (1.04–1.42)	0.015
Yes			1.40 (1.16–1.69)	0.001
Medication use (ref: <5)			1.21 (1.02–1.43)	0.028
Sleep duration (ref: ≥7 hr)				
6.1–6.9 hr			1.23 (1.04–1.45)	0.013
<6 hr			1.45 (1.21–1.73)	<0.001
Excessive daytime sleepiness (ref: Less than a day in a week)			1.27 (1.06–1.52)	0.009
Driving time			1.002 (1.001–1.003)	<0.001
Cognitive impairment (ref: No)			1.08 (0.92–1.28)	0.345

MCR, motoric cognitive risk syndrome; OR, odds ratio; ref, reference; SMC, subjective memory complaints

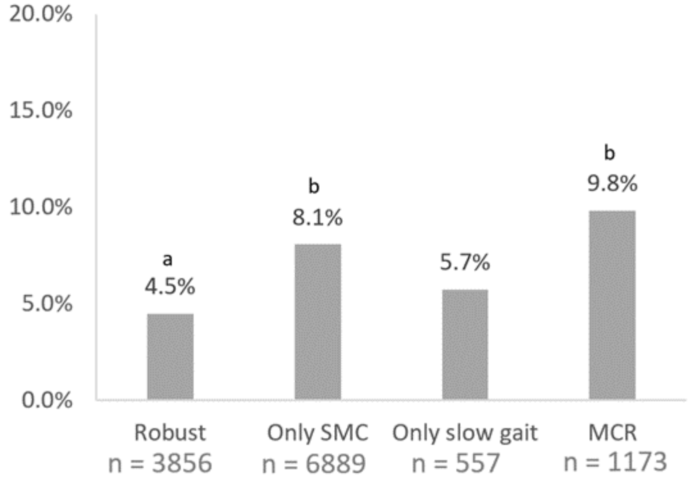

eFigure 1. Comparison of Car Collisions Except Collisions That Were Less Than Half of Driver's Fault Among MCR Assessment Using χ^2 Test

[a] Adjusted standardized residual was > 1.96 ($p < 0.05$).

[b] Adjusted standardized residual was < -1.96 ($p < 0.05$).

© 2023 Kurita S et al. *JAMA Network Open.*

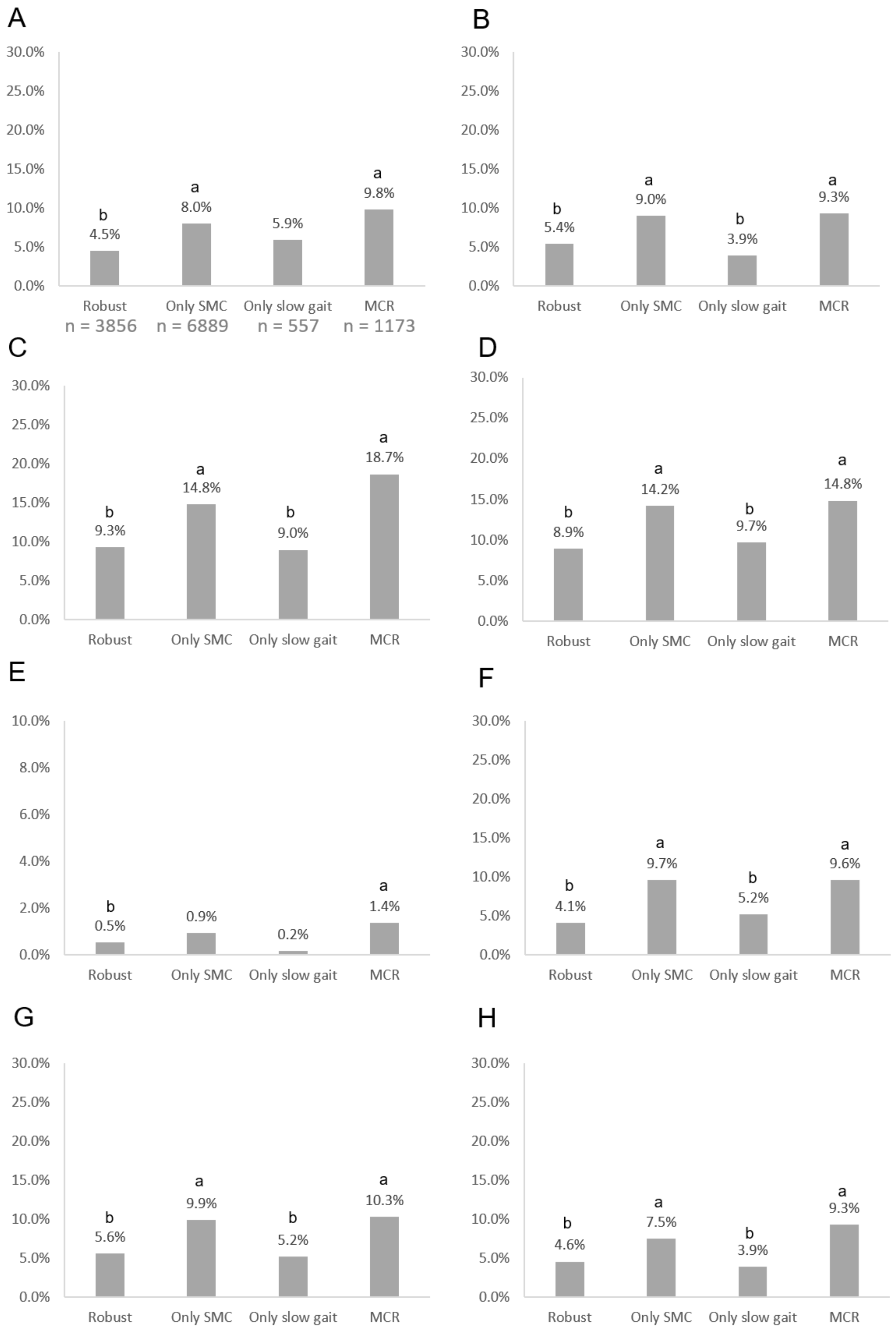

© 2023 Kurita S et al. *JAMA Network Open.*

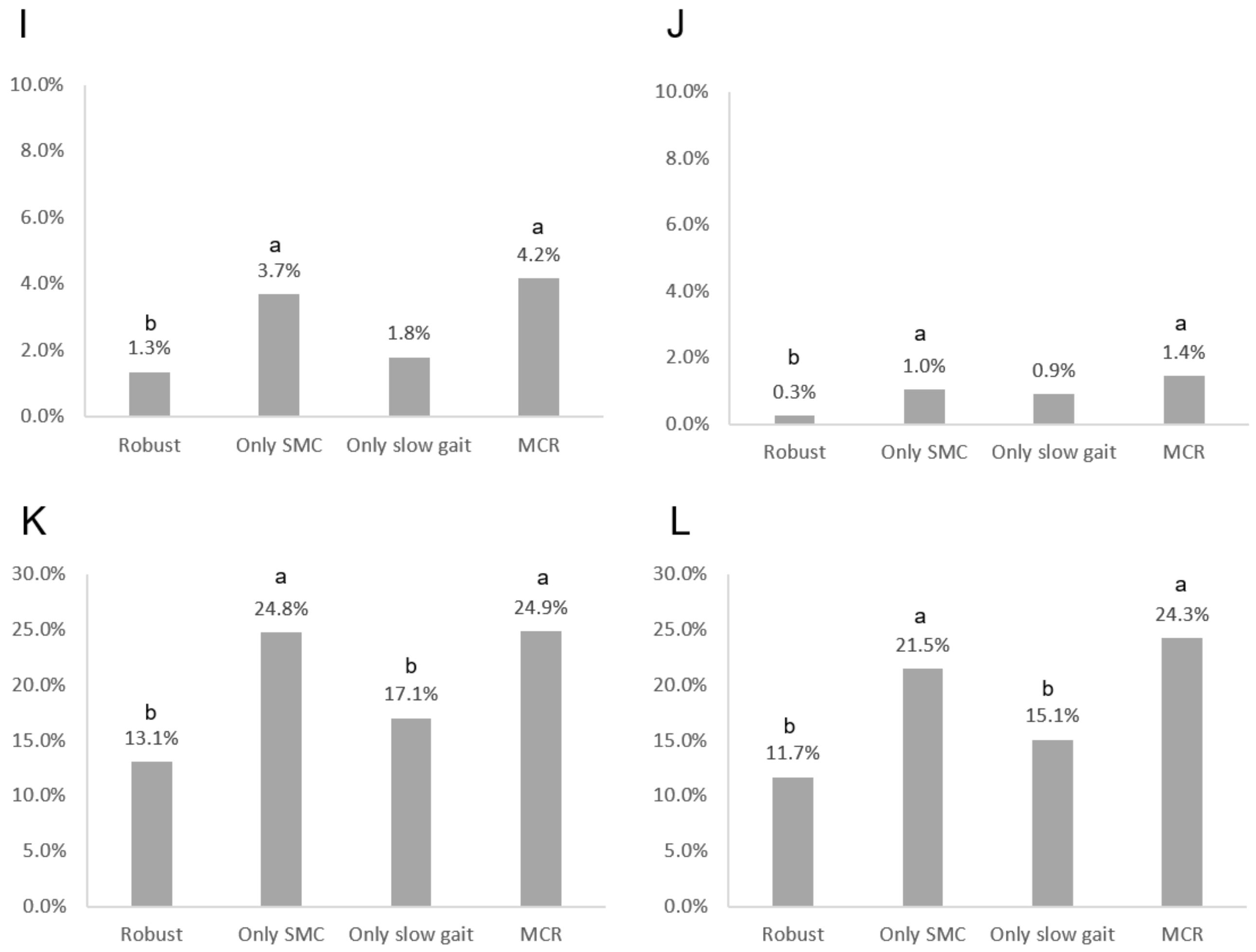

A) When going from a stop line, I almost hit someone coming from a different direction.
B) When attempting to turn right, I almost hit a car coming straight on.
C) When attempting to turn right, I almost hit a pedestrian or bicycle.
D) When attempting to turn left, I almost hit a pedestrian or bicycle.
E) I drifted greatly into the oncoming lane and almost collided head-on with another vehicle.
F) When changing lanes, I almost collided with another vehicle.
G) I almost collided with a vehicle in front of me.
H) I almost struck a bicycle, motorcycle, other vehicle I was overtaking or passing.
I) I made a mistake while stepping on the accelerator or brake.
J) When starting on a hill, I almost hit another vehicle or obstacle (including living things).
K) When backing up to park, I almost hit another vehicle or obstacle (including living things).
L) When I entered a store parking lot from the road, I almost ran up on the curb of the sidewalk.

eFigure 2. Comparison of Each Item of Near-Miss Traffic Incidents Among MCR Assessment Using χ^2 Test

[a] Adjusted standardized residual was > 1.96 ($p < 0.05$).

[b] Adjusted standardized residual was < -1.96 ($p < 0.05$).

© 2023 Kurita S et al. *JAMA Network Open.*

	Participants, n	Events, n	OR (95% CI)
Robust without OCI	3122	140	Ref
Robust with OCI	734	34	1.07 (0.73 - 1.57)
SMC without OCI	5433	435	1.73 (1.42 - 2.11)
SMC with OCI	1456	116	1.75 (1.35 - 2.26)
Slow gait without OCI	403	20	1.07 (0.66 - 1.74)
Slow gait with OCI	154	13	1.93 (1.07 - 3.51)
MCR without OCI	761	70	1.97 (1.46 - 2.67)
MCR with OCI	412	45	2.39 (1.67 - 3.42)

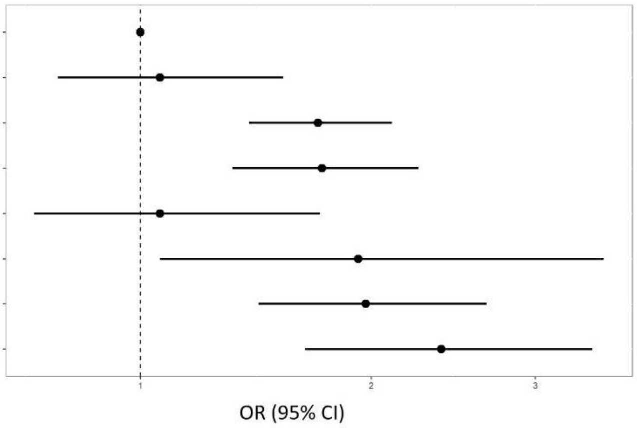

eFigure 3. Odds Ratios of Interactions Between MCR Assessment Findings and OCI on the Associations With Car Collisions Except Those That Were Less Than Half of Driver's Fault

SMC, subjective memory complaints; MCR, motoric cognitive risk syndrome; OCI, objective cognitive impairment

Adjusted for age, sex, educational year, eye diseases, hearing difficulty, medication use, sleep duration, excessive daytime sleepiness, and driving time.

© 2023 Kurita S et al. *JAMA Network Open.*
