# Supplementary material for: Motoric Cognitive Risk Syndrome and Traffic Incidents in Older Drivers in Japan
Source: JAMA Netw Open. 2023 Aug 25;6(8):e2330475. doi: 10.1001/jamanetworkopen.2023.30475 (PMC10457720; doi:10.1001/jamanetworkopen.2023.30475)
Supplement: Supplement 2. — Data Sharing Statement [file jamanetwopen-e2330475-s002.pdf]

Data Sharing Statement

Kurita. Motoric Cognitive Risk Syndrome and Traffic Incidents in Older Drivers in Japan. *JAMA Netw Open*. Published August 25, 2023. doi:10.1001/jamanetworkopen.2023.30475

Data
Data available: No

Additional Information
Explanation for why data not available: The datasets used and/or analyzed during the present study are available from the corresponding author on reasonable request.
